# Clonality of Fluconazole-Nonsusceptible *Candida tropicalis* in Bloodstream Infections, Taiwan, 2011–2017 

**DOI:** 10.3201/eid2509.190520

**Published:** 2019-09

**Authors:** Pao-Yu Chen, Yu-Chung Chuang, Un-In Wu, Hsin-Yun Sun, Jann-Tay Wang, Wang-Huei Sheng, Hsiu-Jung Lo, Hurng-Yi Wang, Yee-Chun Chen, Shan-Chwen Chang

**Affiliations:** National Taiwan University Hospital, Taipei, Taiwan (P.-Y. Chen, Y.-C. Chuang, U.-I. Wu, H.-Y. Sun, J.-T. Wang, W.-H. Sheng, Y.-C. Chen, S.-C. Chang);; National Taiwan University College of Medicine, Taipei (P.-Y. Chen, H.-Y. Wang, Y.-C. Chen, S.-C. Chang);; National Health Research Institutes, Miaoli, Taiwan (J.-T. Wang, H.-J. Lo, Y.-C. Chen)

**Keywords:** candidemia, azoles, drug resistance, multilocus sequence typing, phylogeny, Taiwan, fungi

## Abstract

*Candida tropicalis* is the leading cause of non–*C.* *albicans* candidemia in tropical Asia and Latin America. We evaluated isolates from 344 patients with an initial episode of *C. tropicalis* candidemia. We found that 58 (16.9%) patients were infected by fluconazole-nonsusceptible (FNS) *C. tropicalis* with cross resistance to itraconazole, voriconazole, and posaconazole; 55.2% (32/58) of patients were azole-naive. Multilocus sequence typing analysis revealed FNS isolates were genetically closely related, but we did not see time- or place-clustering. Among the diploid sequence types (DSTs), we noted DST225, which has been reported from fruit in Taiwan and hospitals in Beijing, China, as well as DST376 and DST505–7, which also were reported from hospitals in Shanghai, China. Our findings suggest cross-boundary expansion of FNS *C. tropicalis* and highlight the importance of active surveillance of clinical isolates to detect dissemination of this pathogen and explore potential sources in the community.

*Candida* species are the leading fungal pathogens causing severe healthcare-associated infections in immunocompromised patients globally (*1*). *C. tropicalis* is among the top 4 *Candida* species responsible for candidemia worldwide and is the most common cause of invasive candidiasis in tropical Asia and in Latin America (*2**–**5*).

*C. tropicalis* and *C. albicans* are ascomycetous diploid yeasts, closely related among pathogenic *Candida* species, and belong in a single *Candida* clade characterized by the unique translation of CUG codons as serine rather than leucine (*6*,*7*). These pathogens initially were considered to be susceptible to azoles (*8**–**12*) with the same clinical breakpoints (*13*,*14*). The widespread use of azoles during the past 2 decades coincided with a decrease in incidence of *C. tropicalis* and *C. albicans* infections, which was coupled with an increase in infections caused by *C. glabrata* and other less susceptible and azole-resistant *Candida* species (*12*,*15*,*16*). Azole-resistant, less susceptible *C. tropicalis* has emerged worldwide, particularly in the Asia Pacific region (*5*,*16**–**21*). A multicenter study conducted in this region found that the 90% (MIC_90_) of fluconazole for *C. tropicalis* increased to 32 µg/mL, the same as the MIC_90_ of *C. glabrata* and much higher than the MIC_90_ of *C. albicans*, 0.064 µg/mL (*19*).

Some studies of the genetic relationship of clinical fluconazole-nonsusceptible (FNS) *C. tropicalis* isolates have reported clonal diversity (*22**–**24*), whereas others have demonstrated clonal clusters (*20*,*25*,*26*). Among these studies, few examined the association between genetic relatedness of FNS *C. tropicalis* and clinical characteristics and outcomes of the infected patients (*23*,*27*). We conducted a study of 334 patients with *C. tropicalis* bloodstream infections (BSIs) in Taiwan to examine these relationships in greater detail. We determined the genetic relationships of fluconazole-susceptible (FS) and FNS *C. tropicalis* isolates from blood cultures; compared the relationship of isolates according to time, place, and person; and analyzed the clinical characteristics and outcomes of the patients according to susceptibility to fluconazole and genetic relationship. We further explored the potential emergence and spread of FNS *C. tropicalis* globally.

## Methods

### Study Designs, Setting, and Patients

We conducted a 7-year prospective observational study of adult patients with *C. tropicalis* BSIs admitted to the National Taiwan University Hospital (NTUH; Taipei, Taiwan) during March 1, 2011–December 31, 2017. NTUH is a 2,300-bed teaching hospital that provides both primary and tertiary care. We obtained patient data from the clinical records, including demographics, underlying disease, severity of illness, initial and follow-up blood cultures, focus of infection, antifungal therapy, presence of indwelling catheters, and fatality. We followed patients until discharge or death. This study was approved by the Research and Ethics Committees of NTUH (approval nos. NTUH-201103121RB and NTUH-201502034RIND).

Patients with candidemia were treated according to the guidelines of the Infectious Diseases Society of Taiwan (*28*). Central venous catheters were removed when feasible. Antifungal susceptibility tests were performed at physicians’ request. Systemic antifungal agents included fluconazole, voriconazole, posaconazole, caspofungin, micafungin, anidulafungin, amphotericin B deoxycholate, liposomal amphotericin B, and flucytosine. We defined antifungal exposure as receipt of >1 antifungal agent within 6 months of onset of *C. tropicalis* BSI. We defined breakthrough *C. tropicalis* BSI as a positive blood culture for *C. tropicalis* in patients receiving an antifungal agent for >2 days. 

### Microbiology

We prospectively collected all *C. tropicalis* blood isolates from the hospital clinical microbiology laboratory. We reconfirmed the species identity by using CHROMagar Candida medium (Becton Dickinson, https://www.bd.com) and the Vitek 2 yeast identification system (bioMérieux, https://www.biomerieux.com). We performed DNA sequencing by using the internal transcribed spacer regions of the ribosomal 18S rRNA gene for *Candida* species, as described previously (*29*).

### Antifungal Susceptibility Testing

We determined the MIC of the first *Candida* blood isolate from each patient by using the microdilution colorimetric Sensititre YeastOne YO-09 panel (ThermoFisher Scientific, https://www.thermofisher.com), in accordance with the manufacturer’s instructions. We interpreted MICs according to clinical breakpoints proposed by the Clinical and Laboratory Standards Institute (CLSI) for antifungal agents (*14*); for posaconazole, we used breakpoints proposed by the European Committee on Antimicrobial Susceptibility Testing (EUCAST) (*13*). We defined FS as MIC of ≤2 µg/mL; susceptible-dose-dependent as MIC of 4 µg/mL; and resistant as MIC of ≥8 µg/mL. We further categorized FNS as susceptible-dose-dependent or resistant. We used epidemiologic cutoff values proposed by CLSI to categorize antifungal agents without established clinical breakpoints as wild type or non–wild type (*9*,*30*). We used *C. albicans* (ATCC 90028), *C. parapsilosis* (ATCC 22019), and *C. krusei* (ATCC 6258) as reference strains for quality control. We defined multidrug-resistant (MDR) *C. tropicalis* as nonsusceptible to >1 agent in >2 antifungal classes (*31*).

### DNA Extraction, PCR Amplification, and Sequencing 

We extracted whole-genome DNA of *C. tropicalis* isolates in Sabouraud dextrose agar pure colonies by using Quick-DNA Fungal/Bacterial DNA MiniPrep Kit (Zymo Research, https://www.zymoresearch.com) according to manufacturer’s protocol. We measured DNA concentrations by using NanoDrop 2000 (ThermoFisher Scientific, https://www.thermofisher.com). We stored DNA extracts at −20°C before conducting amplification in a reaction volume of 20 μL, consisting of 2 μL of DNA, 1 μL each of forward and reverse primers (50 mmol/L), 10 μL KAPA HotStart ReadyMix (KAPA Biosystems, https://www.kapabiosystems.com), and 6 μL of water. We performed PCR amplification by the following methods: 95°C for 3 min; 30 cycles of 95°C for 30 sec, 60°C for 30 sec, and 72°C for 30 sec; 72°C for 3 min; and a final hold at 4°C.

### Multilocus Sequence Typing

We typed all FNS isolates and randomly selected FS isolates to type at a ratio of 1:2 a multilocus sequence typing (MLST) scheme previously described by Tavanti et al (*32*). In brief, we used sequences of the oligonucleotide primers for PCR amplification of 6 gene fragments, *ICL1*, *MDR1*, *SAPT2*, *SAPT4*, *XYR1*, and *ZWF1a*. We purified the PCR amplification products and sequenced both strands of the fragments by using an Applied Biosystems PRISMR 3730 DNA Analyzer (ThermoFisher Scientific, https://www.thermofisher.com). We defined nucleotide sequences by alignment of forward and reverse sequences by using BioNumerics version 6.6 (Applied Maths, http://www.applied-maths.com) and confirmed polymorphic sites by visual examination of the chromatograms. We defined heterozygosity as the presence of 2 coincident peaks in both the forward and reverse sequence chromatograms. We defined the results by using heterozygous data (K, M, R, S, W, and Y) from the International Union of Pure and Applied Chemistry (https://iupac.org) nomenclature.

To assign allele numbers and diploid sequence types (DSTs), we compared our sequences with *C. tropicalis* available in the central MLST database (https://pubmlst.org/ctropicalis) and assigned new allele numbers as needed. We used a combination of the results from the 6 gene fragments that yielded unique DSTs to quantify the similarities and putative genetic relationships between *C. tropicalis* isolates.

### Phylogenetic Analysis

We conducted phylogenetic analysis by the UPGMA and applied minimum spanning tree algorithms based on p-distance by using BioNumerics version 6.6 to concatenated sequence data of 165 *C. tropicalis* isolates in this cohort. Only 55 FNS isolates were typable. We determined the value of the cluster nodes by bootstrapping with 1,000 randomizations and used eBURST V3 (Imperial College London, UK; http://eburst.mlst.net) to determine putative relationships between strains. When 5 of the 6 alleles were identical between a pair, we considered the strains related and placed them into clonal complexes (CCs) (*32*). We predicted the putative founding DST of each CC by using the eBURST algorithm, where possible.

We downloaded 185 FNS *C. tropicalis* isolates from the MLST database and included these for phylogenetic analyses to elucidate the global clonal spread of these fungi. We also reviewed the MLST database and the literature to identify the year, country, and city from which these isolates were reported or detected.

### Data Analysis

We expressed continuous variables as medians and interquartile ranges, and categorical variables as absolute frequencies and percentages. To compare clinical and microbiological factors between FS and FNS groups, we analyzed continuous data with the Mann-Whitney U test. We compared categorical data using χ^2^ or Fisher exact test. For 3 groups categorized by CCs and FS, we performed post hoc analysis using the Mann-Whitney U test or Fisher exact test with a Bonferroni-adjusted α for pairwise comparisons if the result of the initial test was statistically significant. We examined time trends of the rates by logistic regression analysis. To analyze the predictors of FNS *C. tropicalis* BSIs, we subsequently entered all variables with p<0.20 in univariate analysis and probable biologic meaning into the multivariate analysis. We developed multivariate models by using a stepwise method, with minimization of the Akaike information criterion, then considered variables statistically significant only when p<0.05 and included these in the final model. We performed analyses with Stata version 14 (StataCorp LLC, https://www.stata.com) software and considered 2-sided p values <0.05 statistically significant.

## Results

### *C. tropicalis* Susceptibility 

We compiled in vitro susceptibility profiles to 9 antifungal drugs for 344 initial *C. tropicalis* blood isolates (Table 1). We found 58 (16.9%) isolates that were either FS (48/344, 14.0%) or susceptible-dose-dependent (10/344, 2.9%). We noted some differences in susceptibility to other azoles. Two isolates were resistant to 3 echinocandins, and all were susceptible to amphotericin B. Overall, only 1 isolate was categorized as MDR to all tested azoles and echinocandins. 

**Table 1:** Comparison of antifungal susceptibility distribution of 344 *Candida troplicalis* blood isolates, Taiwan, 2011–2017*

Antifungal agents	Total, n = 344	Fluconazole-susceptible isolates, n = 286	Fluconazole-nonsusceptible isolates		
			Total,† n = 58	Clonal complex 3,‡ n = 36	Other clonal complexes,‡ n = 19
Fluconazole					
MIC_50_	1	1	216	256	8
MIC_90_	32	2	512	512	128
Range	0.06–512	0.06–2	4–512	32–512	4–512
NS rates, no. (%)	58 (16.9)	0	58 (100)§	36 (100)	19 (100)
Itraconazole					
MIC_50_	0.25	0.25	0.5	0.5	0.06
MIC_90_	0.5	0.25	1	1	1
Range	0.06–32	0.03–0.5	0.06–32	0.25–1	0.06–32
NWT rates, no. (%)	20 (5.8)	0	20 (34.5)§	18 (50)	2 (10.5)
Posaconazole					
MIC_50_	0.25	0.25	0.5	0.5	0.5
MIC_90_	0.5	0.5	1	1	1
Range	0.06–16	0.004–0.5	0.06–16	0.25–2	0.06–16
NS rates, no. (%)	285 (82.9)	228 (79.7)	57 (98.3)§	36 (100)	18 (94.7)
Voriconazole					
MIC_50_	0.12	0.12	4	8	0.5
MIC_90_	2	0.12	16	16	4
Range	0.004–16	0.004–0.25	0.25–16	1–16	0.25–16
NS rates, no. (%)	75 (21.8)	17 (5.9)	58 (100)§	36 (100)	19 (100)
Anidulafungin					
MIC_50_	0.06	0.06	0.12	0.12	0.06
MIC_90_	0.12	0.12	0.12	0.12	0.12
Range	0.008–1	0.008–0.5	0.008–1	0.008–1	0.008–0.25
NS rates¶, no. (%)	2 (0.6)	1 (0.4)	1 (1.7)	1 (2.8)	0
Caspofungin					
MIC_50_	0.06	0.06	0.06	0.06	0.06
MIC_90_	0.12	0.12	0.25	0.25	0.25
Range	0.015–8	0.015–2	0.015–8	0.015–8	0.015–0.25
NS rates¶, no. (%)	3 (0.9)	2 (0.7)	1 (1.7)	1 (2.8)	0
Micafungin					
MIC_50_	0.03	0.03	0.03	0.03	0.03
MIC_90_	0.03	0.03	0.03	0.03	0.03
Range	0.015–2	0.004–1	0.015–2	0.015–2	0.015–0.03
NS rates¶, no. (%)	2 (0.6)	1 (0.4)	1 (1.7)	1 (2.8)	0
Amphotericin					
MIC_50_	1	1	1	1	1
MIC_90_	1	1	1	1	1
Range	0.25–1	0.25–1	0.25–1	0.50–1	0.25–1
NWT rates, no. (%)	0	0	0	0	0
Flucytosine					
MIC_50_	0.03	0.03	0.03	0.03	0.03
MIC_90_	0.06	0.06	0.12	0.03	0.50
Range	0.03–64	0.03–64	0.03–32	0.03–0.03	0.03–32
NWT rates, no. (%)	4 (1.2)	3 (1.0)	1 (1.7)	0	1 (5.3)

*MICs and ranges are reported in μg/mL. NS, nonsusceptible; NWT, non–wild-type. †Of 58 fluconazole-nonsusceptible isolates, only 55 isolates were typable with subsequent assignment of clonal complex. ‡Details of CCs and corresponding MIC data are available in Appendix Table 1 (https://wwwnc.cdc.gov/EID/article/25/9/19-0520-App1.pdf). §Comparison of antifungal NS rates between FS isolates (n = 256) and FNS isolates (n = 58) by χ^2^ tests, p<0.001. ¶The susceptibility discrepancy among 3 echinocandins may be attributed to significant variability in caspofungin susceptibility testing, which resulted in false resistance reporting.

**Figure 1:**
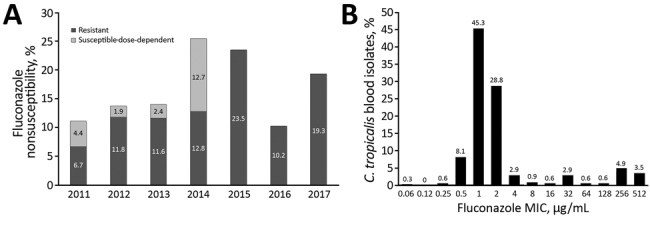
Fluconazole nonsusceptiblity of *Candida tropicalis* blood isolates, Taiwan, 2011–2017. A) Proportions of fluconazole nonsusceptibility among 344 *C. tropicalis* blood isolates by year. B) Distributions of fluconazole MICs among *C. tropicalis* blood isolates.

We also compiled annual FNS rates for all *C. tropicalis* isolates during 2011–2017 (Figure 1, panel A). Of note, the rate of resistance to fluconazole increased from 6.7% in 2011 to 19.3% in 2017 (p = 0.07 for the trend). The distribution of fluconazole MICs was bimodal. The highest peak ranged from 0.5 to 2 µg/mL, with a smaller peak at 128–512 µg/mL (Figure 1, panel B).

### Phylogenetic Analysis of the *C. tropicalis* Blood Isolates

The UPGMA dendrogram for 55 FNS and 110 FS isolates (Appendix Figure ) showed that the 165 isolates belonged to 16 groups defined by similarities of >80% and consisting of 87 DSTs. eBURST analysis revealed 78 DSTs grouped into 24 CCs; 9 DSTs were classified as singletons (Appendix Figure 1, Appendix Table 1). The CCs determined by eBURST were concordant to groups defined by UPGMA, except for some minor CCs and singletons that were grouped with other major CCs by UPGMA. CC3 was most common (40/165, 24.2%), correlating with UPGMA group 9 with 85.2% similarity; isolates were assigned to 12 DSTs, including DST225 (n = 9) as the putative founder based on eBURST algorithm. CC2 was the second most common, correlating with UPGMA group 4; these 33 isolates were assigned to 13 DSTs, with DST140 (n = 14) as the putative founder. CC4, UPGMA group 7, included 22 isolates assigned to 12 DSTs, with DST139 (n = 7) as the putative founder.

CC3 had an FNS rate of 90.0% (36/40) compared with variable rates for the other CCs; 65.5% (36/55) of the FNS isolates from this study belonged to CC3, including DST225 (n = 9), DST375 (n = 1), DST376 (n = 6), DST505 (n = 1), DST506 (n = 6), DST507 (n = 10), DST753 (n = 1), DST754 (n = 1), and DST838 (n = 1). A minor cluster of FNS isolates belonged to CC11, including DST508 (n = 3) and DST752 (n = 1). The remaining18 FNS isolates were scattered among 11 different CCs or singletons. We found similar genetic relationships with other azoles, but no correlation of genetically similar isolates with time or place, or clustering of the cases in the hospital (Appendix Figure 1).

### Genetic Relationships of FNS *C. tropicalis*

**Figure 2:**
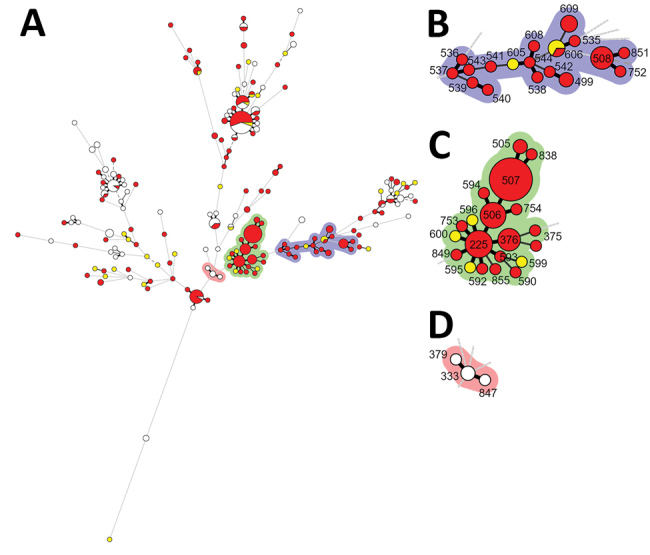
Minimum spanning tree of 350 *C. tropicalis* isolates from multilocus sequence typing (MLST) data. A) Minimum spanning tree of 165 *C. tropicalis* blood isolates from this study’s cohort (Taiwan, 2011–2017) and 185 isolates with fluconazole nonsusceptibility from the central *C. tropicalis* MLST global database (https://pubmlst.org/ctropicalis). Each circle corresponds to a diploid sequence type (DST). The size of the circle indicates the number of the isolates belonging to a specific DST and classified as fluconazole resistant (red), susceptible-dose-dependent (yellow), or susceptible (white). Lines between circles indicate the similarity between profiles: bold lines indicate 5 of 6 alleles are identical, solid lines indicate 4 alleles are identical, and dotted lines indicate ≤3 alleles are similar. Shaded areas indicate groups of target clonal complexes (CCs). B) Enlarged area of CC10 and CC11 (purple shading). C) Enlarged area of fluconazole nonsusceptible CC3 (green shading). D) Enlarged area of fluconazole susceptible CC3 (pink shading).

We further evaluated the genetic relationships of 165 *C. tropicalis* blood isolates from our cohort with 185 FNS strains available in the MLST database. The minimum spanning tree of the 350 isolates is composed largely of CC3, CC10, and CC11 with high FNS rates (Figure 2) that share 78.0% similarity on the basis of the UPGMA algorithm (Appendix Figure 2). 

We also summarized the year of isolation, country and city of origin, and clinical or environmental sites of *C. tropicalis* CC3, CC10, and CC11, all of which were reported from countries in Asia, most after 2011 (Appendix Table 2). CC3 again formed the largest cluster; 22 DSTs, including DST225, were isolated from the environment and hospitals in Taiwan. DST225 also was isolated in hospitals in Beijing, China. DST375 and DST505–7 were isolated in hospitals in Shanghai, China. CC10 was the second largest cluster with 12 DSTs reported from Singapore and Nanchang, China, but we did not find these in our study. CC11 with 5 DSTs was reported from Singapore and China (Beijing, Shanghai, and Nanchang). DST508 was isolated in the current study in Taiwan and Beijing.

### Clinical Characteristics and Outcomes of Patients with *C. tropicalis* BSIs

Of the 58 patients in this study with FNS isolates, 32 (55.2%) had no previous antifungal exposure (Table 2, Appendix Table 3). Nevertheless, multivariate logistic regression analysis revealed that antifungal drug exposure was associated with FNS infection (odds ratio [OR] 5.64, 95% CI 2.94–10.81; p<0.001). Another risk factor for FNS infection was moderate to severe liver disease (adjusted OR 3.13, 95% CI 1.06–9.24; p = 0.04). We saw no statistically significant difference in deaths or persistent candidemia between patients according to the degree of fluconazole susceptibility of their isolates.

**Table 2:** Comparisons of clinical and microbiological characteristics between fluconazole-susceptible and fluconazole-nonsusceptible *Candida tropicalis* bloodstream infections, Taiwan, 2011–2017*

Characteristic	Total, n = 344	With FS *C. tropicalis* BSIs, n = 286	With FNS *C. tropicalis* BSIs, n = 58	p value
Demographics				
Age, y, median (IQR)	62.8 (53.2–73.5)	62.4 (53.0–74.3)	63.4 (55.2–72.1)	0.85
Sex, no. (%)				0.54
M	201 (58.4)	165 (57.7)	36 (62.1)	
F	143 (41.6)	121 (42.3)	22 (37.9)	
Disease severity				
ICU onset, no. (%)	105 (30.7)	85 (29.9)	20 (34.5)	0.49
APACHE II score, median (IQR)	20.0 (15.0–26.0)	20.0 (15.0–26.0)	19.0 (15.5–26.0)	0.85
Healthcare factors, no. (%)†				
Solid organ transplant	4 (1.2)	3 (1.1)	1 (1.8)	0.52
Hematopoietic stem cell transplant	10 (2.9)	9 (3.2)	1 (1.8)	0.99
Major surgery	40 (11.6)	34 (11.9)	6 (10.3)	0.99
Parenteral hyperalimentation	189 (59.4)	155 (54.2)	34 (58.6)	0.54
Steroid use	170 (49.4)	133 (46.5)	37 (63.8)	0.02
Chemotherapy	153 (44.5)	123 (43.0)	30 (51.7)	0.22
Neutropenia	91 (26.8)	69 (24.5)	22 (38.6)	0.03
Mechanical ventilator	101 (29.4)	84 (29.4)	17 (29.3)	0.99
Indwelling urinary catheter	138 (40.1)	110 (38.5)	28 (48.3)	0.16
Central venous catheter	286 (83.1)	238 (83.2)	48 (82.8)	0.93
Antifungal exposure	60 (17.4)	34 (11.9)	26 (44.8)	<0.001
Antibiotics exposure	300 (87.7)	248 (87.3)	52 (89.7)	0.62
Therapeutic intervention, no. (%)‡				
Early appropriate antifungal agents	261 (75.9)	243 (85.0)	18 (31.0)	<0.001
Fluconazole as the first antifungal agent	221 (64.2)	185 (64.7)	36 (62.1)	0.71
Early removal of central venous catheter	162/286 (56.6)	131/238 (55.0)	31/48 (64.6)	0.22
Clinical outcomes, no. (%)				
Death				
7 d	73 (21.2)	60 (21.0)	13 (22.4)	0.81
14 d	117 (34.0)	99 (34.6)	18 (31.0)	0.60
28 d	167 (48.6)	141 (49.3)	26 (44.8)	0.53
In hospital	226 (65.7)	187 (65.4)	39 (67.2)	0.79
Persistence, no. (%)§	81 (27.7)	65 (26.6)	16 (33.3)	0.34

*Additional information on patient conditions and microbiological data can be found in Appendix Table 3 (https://wwwnc.cdc.gov/EID/article/25/9/19-0520-App1.pdf). APACHE, Acute Physiology and Chronic Health Evaluation; BSIs, bloodstream infections; FNS, fluconazole nonsusceptible; FS, fluconazole susceptible; ICU, intensive care unit; IQR, interquartile range.  †Major surgery refers to cardiovascular or abdominal surgery. Classes of antifungal exposure to azole or echinocandin, 31/3 in FS group vs. 24/2 in FNS group; of note, 14 (24.1%) patients in the FNS group experienced breakthrough bloodstream infections, compared with 18 (6.3%) patients in the FS group (p<0.001). ‡Early adequate antifungal agents refers to administration of the recommended dose of an intravenous antifungal agent within 48 h after first positive blood culture collection for a susceptible *Candida* isolate, according to the Clinical and Laboratory Standards Institute (CLSI) species-specific breakpoints (*14*). Early removal of central venous catheters is defined as removal of all similar devices, including tunneled and peripherally inserted central catheters, within 48 h after obtaining the first positive blood culture. §Persistence is defined as >5 days of blood cultures positive for the same *Candida* species.

We divided the 165 initial blood isolates with known DSTs into 3 groups, FNS CC3, FNS other CCs, and FS isolates (Appendix Table 4). Patients infected with FNS CC3 were more likely to have neutropenia, previous steroid use, and chemotherapy by Fisher exact test with a Bonferroni adjustment; two thirds previously were exposed to antifungal drugs. We saw no statistically significant difference in outcome among patients from the 3 groups.

## Discussion

In our 7-year observational study in Taiwan, we found an increasing trend over time in the emergence of fluconazole-resistant *C. tropicalis* isolates in blood. Over time, fluconazole susceptible-dose-dependent *C. tropicalis* strains were replaced by fully resistant strains. Many of the FNS isolate strains were genetically closely related to each other and to strains from the environment and other hospitals in Taiwan and other countries in Asia. We found a 6-fold increase in the risk for FNS *C. tropicalis* infection in patients with prior exposure to antifungal drugs, but half of the FNS isolates we obtained were from azole-naive patients. We saw no statistically significant relationship of the cases to time and place and no clustering.

The drive of antifungal resistance is most commonly attributed to antifungal selection pressure, especially in human use (*23*). Our finding of an increased risk for *C. tropicalis* BSIs in patients who received antifungal drugs in the preceding 6 months supports this scenario. Furthermore, our study showed a high rate (14/26, 53.8%) of breakthrough BSIs (receipt of antifungal drugs >2 days before BSI onset) among FNS isolates from patients with antifungal exposure. These breakthrough isolates showed higher fluconazole MIC_50_ (256 μg/mL) than isolates with only prior exposure to antifungal agents (32 μg/mL). This result reflected higher antifungal selection pressure during current antifungal use than with previous use only.

Alternatively, azole resistance in human fungal pathogens might develop through exposure to azole fungicides in the environment. Our nationwide environmental surveillance and multicenter clinical study is concordant with global concerns that azole resistance in *A. fumigatus* human isolates, at least in part, resulted from resistant strains in the environment and the use of azole fungicides in agricultures (*33*,*34*). According to that study, annual consumption of 5 fungicide azoles in Taiwan increased 4-fold during 2003–2016, indicating long-existing high fungicide burdens in the environment in Taiwan during our study period (*34*).

Meanwhile, nationwide environmental surveys in Taiwan isolated *C. tropicalis* DST225 from fruit (*35*) and from patients in different hospitals enrolled in the Taiwan Surveillance of Antimicrobial Resistance of Yeasts (*35*). DST225 isolates in that investigation showed cross resistance to fluconazole and triadimenol, an azole fungicide (*35*). Given that DST225 and genetically related DSTs were identified in clinical isolates obtained from azole-naive patients in our study and in a report from China (*27*), along with high fungicide burden in Asia (*36*), we suggest that patients could acquire FNS *C. tropicalis* from the environment in the community. FNS *C. tropicalis* without time- and place-clustering in this study further excludes the potential for cross-transmission in hospitals.

We propose the multifocal emergence of genetically related FNS *C. tropicalis* strains in Taiwan and other countries in Asia (*25**–**27*) is a result of the selective pressure of intense use of azole antifungal agents in humans and agriculture (*34**–**36*). Furthermore, human use promotes the selection of resistant strains in patients already colonized from environmental sources by susceptible-dose-dependent or resistant genotypes of *C. tropicalis*. It is unclear whether these strains arise independently or are spread by extensive trade of agricultural products among these countries.

In our study, FNS isolates were not associated with worse clinical and microbiological outcomes. This finding was concordant with prior studies demonstrating no good correlations between outcomes of patients with *Candida* BSIs and fluconazole MIC or pharmacodynamics parameters, such as the area under the concentration-time curve to MIC ratio (*11*,*18*,*37*).

The strength of this study is that it used a large cohort of *C. tropicalis* blood isolates collected over a 7-year period and integrated with the *C. tropicalis* MLST central database and other published data through literature review and to infer the genetic relationships of FNS *C. tropicalis* globally (Appendix Table 2). However, this study has several limitations. It was conducted in a single hospital in Taiwan, and one should be cautious in making generalizations because differences in nosocomial spread might occur in other institutions. We likely underestimate the proportion of FNS *C. tropicalis* in this cohort because the study focused on isolates from blood and was limited to the first episode of *C. tropicalis* BSI from each patient. Furthermore, we did not define the mechanisms for development of resistance, which were previously examined in China and Singapore. Other studies have shown that *ERG*11 mutations combined with or without *MDR*1 overexpression produce high-level resistance to fluconazole and other azoles in *C. tropicalis* isolates belonging to CC3, CC10, and CC11, consisting of DST225 and genetically related DSTs (*26*,*27*).

In conclusion, FNS *C. tropicalis* clones appear to have emerged in part due to use of azole antifungal agents in agriculture, with cross-country expansion fostered by therapeutic use in hospitals. The concept that FNS *C. tropicalis* was acquired outside the hospital is supported by the lack of evidence of nosocomial spread. These findings emphasize the importance of active surveillance of FNS *C. tropicalis* in agriculture, hospitals, and the community.

Appendix Additional information on clonality of fluconazole-nonsusceptible *Candida tropicalis* in bloodstream infections, Taiwan, 2011–2017.
