# Supplementary material for: Clonality of Fluconazole-Nonsusceptible Candida tropicalis in Bloodstream Infections, Taiwan, 2011–2017
Source: Emerg Infect Dis. 2019 Sep;25(9):1668–75. doi: 10.3201/eid2509.190520 (PMC6711239; doi:10.3201/eid2509.190520)
Supplement: Appendix — Additional information on clonality of fluconazole-nonsusceptible Candida tropicalis in bloodstream infections, Taiwan, 2011–2017. [file 19-0520-Techapp-s1.pdf]

Article DOI: https://doi.org/10.3201/eid2509.190520

Clonality of Fluconazole-Nonsusceptible *Candida tropicalis* in Bloodstream Infections, Taiwan, 2011–2017

Appendix

Appendix Table 1. Distribution of 24 clonal complexes and 9 singletons with corresponding diploid sequence types and fluconazole susceptibility test results among 165 *C. tropicalis* blood isolates, Taiwan, 2011–2017*

Clonal complex†	DST, no. (n = 87)	Isolates, no. (%) (n = 165)	Diploid sequence type	Fluconazole		
				MIC_{50} (µg/mL)	MIC_{90} (µg/mL)	Nonsusceptible, no. (%)
3	12	40 (24.2)	**507** (10), **225** (9), **376** (6), **506** (6), 333 (2), **375** (1), 379 (1), **505** (1), **753** (1), **754** (1), **838** (1), 847 (1)	256	512	36 (90.0)
2	13	33 (20.0)	140 (14), **98** (5), 144 (3), 357 (2), **45** (1), 168 (1), 388 (1), 389 (1), 820 (1), 829 (1), 830 (1), **858** (1), 859 (1)	2	2	3 (9.1)
4	12	22 (13.3)	139 (7), 171 (2), 184 (2), 823 (2), 833 (2), 257 (1), 386 (1), 558 (1), **757** (1), 825 (1), 832 (1), 834 (1)	1	2	1 (4.6)
Singleton	9	10 (6.1)	826 (2), **387** (1), **758** (1), 822 (1), 824 (1), 827 (1), 828 (1), 836 (1), 837 (1)	1	2	2 (20.0)
1	7	10 (6.1)	384 (3), 134 (2), 90 (1), 203 (1), 377 (1), 563 (1), 821 (1)	1	2	0
22	4	9 (5.5)	**183** (6), 361 (1), 517 (1), 831 (1)	1	2	1 (11.1)
9	1	4 (2.4)	169 (4)	1	2	0
11	2	4 (2.4)	**508** (3), **752** (1)	16	32	4 (100)
36	4	4 (2.4)	364 (1), 365 (1), 366 (1), 367 (1)	0.25	1	0
8	2	3 (1.8)	359 (2), 549 (1)	1	2	0
20	3	3 (1.8)	373 (1), 817 (1), 839 (1)	1	1	0
35	2	3 (1.8)	**374** (2), 818 (1)	2	4	1 (33.3)
50	2	3 (1.8)	369 (2), **378** (1)	2	4	1 (33.3)
6	2	2 (1.2)	149 (1), 835 (1)	2.0	2.0	0
14	2	2 (1.2)	**587** (1), **846** (1)	4.0	4.0	2 (100)
28	1	2 (1.2)	571 (2)	0.5	2	0
40	1	2 (1.2)	187 (2)	1	1	0
49	1	2 (1.2)	358 (3)	0.5	0.5	0
5	1	1 (0.6)	**394** (1)	4	NA	1 (100)
25	1	1 (0.6)	**525** (1)	4	NA	1 (100)
34	1	1 (0.6)	564 (1)	2	NA	0
47	1	1 (0.6)	**370** (1)	128	NA	1 (100)
58	1	1 (0.6)	**164** (1)	8	NA	1 (100)
62	1	1 (0.6)	165 (1)	2	NA	0
63	1	1 (0.6)	172 (1)	0.5	NA	0

*The order of diploid sequence types in each clonal complex are listed by numbers of isolates per DST; DST numbers are given from lowest to highest. DST numbers with bold indicate fluconazole nonsusceptibility, but a DST can contain both fluconazole nonsusceptible (FNS) and fluconazole susceptible (FS) isolates. CC, clonal complex; DST, diploid sequence type; MIC_{50}, minimum inhibitory concentration encompassing 50% of isolates; MIC_{90}, minimum inhibitory concentration encompassing 90% of isolates; NA, not applicable.
†For CCs with <5 isolates, minimum inhibitory concentration 50 (MIC_{50}) is replaced by minimum MIC and MIC_{90} is replaced with maximum MIC. For isolates with only 1 CC, MIC_{50} is replaced with exact MIC and MIC_{90} is not applicable.

Appendix Table 2. Summary of the year and location in which selected genotypes of *Candida tropicalis* clinical and environment isolates from clonal complex 3, 10, and 11 were reported*

Clonal complex	DST	Year	Location	Source	Isolate number	Fluconazole MIC (µg/mL)	References
**3**	225	2012–2016	Taiwan, NTUH	C	F2012i031, F2013 g040, F2014 g069, F2015f002, F2015f066, F2016a011, F2016a073, F2016d089, F2016e086	32–512	This study
		2012	Taiwan, Others	E	F85	4	*4*
		NA	Taiwan, Others	C	–	–	*4*
		2014–2015	Beijing, China	C	Ct01R, Ct07R, Ct08R, Ct09R	64–>256	*5*
	287	2010	Beijing, China	C	BZR-62	–	*1*
	294	2011	Beijing, China	C	BZR-71	–	*1*
	330	2009, 2010	Chengdu, China	C	09HX010, 10HX073	–	*1*
		N/A	Hainan, China	C	DZ29_hn_m_8_Pt	–	*1*
		2011, 2014	Shenzhen, China	C	F20, E616	–	*1*
	333	2014, 2017	Taiwan, NTUH	C	F2014e092, F2017f024	1	This study
		2010	Chengdu, China	C	10HX030	–	*1*
		N/A	Hainan, China	–	DFR39_hn_m_8_Pt	–	*1*
		2006, 2011	Shenzhen, China	C	G213, E446	–	*1*
		2015–2017	Italy	C	IRCCS-3, IRCCS-40, IRCCS-42, IRCCS-43, B4BR3, ALOG1	<1	*6*
	375	2013	Taiwan, NTUH	C	F2013a028	–	This study, *1*
	376	2013–2017	Taiwan, NTUH	C	F2012f046, F2012f083, F2013a013, F2015c056, F2017c014, F2017c078	64–512	This study, *1*
		2012–2015	Shanghai, China	C	RC51, RC165, RC181, RC193, RC289, RC366, RC519, RC527	16–168	*2*
	379	2011	Taiwan, NTUH	C	F2011ah028	1	This study, *1*
	411	2010	Beijing, China	C	ZRCT27	–	*1*
	426	2012	Gwangju, South Korea	C	BS48	–	*1*
		2008	Shenzhen, China	C	8559	–	*1*
	449	2016	Hainan, China	C	SYL5_2_14_hn_f_65_Pt	–	*1*
	505	2017	Taiwan, NTUH	C	F2017e079	512	This study
		2012	Shanghai, China	C	572	64	*1,2*
	506	2014–2015	Taiwan, NTUH	C	F2014f079, F2014 g052, F2015a060, F2015d058, F2015e040, F2015e098	256–512	This study
		2014	Taiwan, Others	C	YM140066	64	*1*
		2013	Shanghai, China	C	573	64	*1,2*
		2015	Beijing, China	C	Ct10R	>256	*5*
	507	2013–2017	Taiwan, NTUH	C	F2013h050, F2015a021, F2015d026, F2016a057–2, F2017b087, F2017d061, F2017e044, F2017f090, F2017 g013, F2017 g048	128–512	This study
		2013–2015	Shanghai, China	C	574, 575, 576, 577, 578, 579, 580, 581, 582, 583, 584, 585, 586	32–128	*1,2*
	532	2014	Shanghai, China	C	618	1	*1*
		2011, 2013	Shenzhen, China	C	E303, T083	–	*1*
	546	2014	Taiwan, Others	C	F2014f003	–	*1*
	590	2014	Taiwan, Others	C	YM140663	32	*1*
	592	2012	Taiwan, Others	–	YFA123445	64	*1*
	593	2014	Taiwan, Others	C	YM140586	64	*1*
	594	2014	Taiwan, Others	C	YM140285	64	*1*
	595	2014	Taiwan, Others	C	YM140907	4	*1*
	596	2012	Taiwan, Others	–	YFA121135	4	*1*
	599	2014	Taiwan, Others	–	YM140982	4	*1*
	600	2014	Taiwan, Others	C	YM140789	4	*1*
	724	2008	Shenzhen, China	C	8152	–	*1*

Clonal complex	DST	Year	Location	Source	Isolate number	Fluconazole MIC (µg/mL)	References
	753	2012	Taiwan, NTUH	C	F2012d069	64	This study
	754	2013	Taiwan, NTUH	C	F2013e089	256	This study
	838	2017	Taiwan, NTUH	C	F2017f039	256	This study,*1*
	847	2017	Taiwan, NTUH	C	F2017 g035	2	This study
	849	2013	Taiwan, Others	–	CT105	64	*1*
	855	2012	Taiwan, Others	–	CT261	128	*1*
**10**	499	2014	Singapore	C	K2, 624	>256	*1,3*
	536	2014	Singapore	C	623	>256	*1,3*
	537	2014	Singapore	C	625	64	*1,3*
	538	2014	Singapore	C	626	>256	*1,3*
	539	2014	Singapore	C	627	256	*1,3*
	540	2014	Singapore	C	628	>256	*1,3*
	541	2014	Singapore	C	629	96	*1,3*
	542	2014	Singapore	C	630	>256	*1,3*
	543	2014	Singapore	C	631	>256	*1,3*
	544	2014	Singapore	C	632	>256	*1,3*
	545	2014	Singapore	C	633	0.25	*1,3*
	605	2016	Nanchang, China	C	9238	4	*1*
	608	2016	Nanchang, China	C	12070	64	*1*
**11**	335	2010	Chengdu, China	C	10HX044	–	*1*
	508	2014–2017	Taiwan, NTUH	C	F2014f021, F2015c031, F2017b083	16–32	This study
		2013–2015	Shanghai, China	C	587, 588, 589	8–16	*1,2*
		2014	Shenzhen, China	–	F291	–	*1*
		2015	Beijing, China	C	Ct11R, Ct12R	64	*5*
	521	2014	Shanghai, China	C	605	1	*1*
	535	2015	Singapore	C	622	>256	*1,3*
	606	2016	Nanchang, China	C	10307, 10285, 10471	4–8	*1*
	609	2016	Nanchang, China	C	10787–2, 12127, 10787–1	8	*1*
	610	2016	Nanchang, China	C	10215	2	*1*
	752	2014	Taiwan, NTUH	C	F2014d080	32	This study
	843	2012	Taiwan, Others	C	C2–1010802	–	*1*
	851	2014	Taiwan, Others	–	CT152	16	*1*

*Only fluconazole nonsusceptible isolates obtained from the *Candida tropicalis* multilocus sequence typing (MLST) central database (https://pubmlst.org/ctropicalis) were added to 165 blood isolates in the current cohort for phylogenetic analyses in Figure 2 panels A and B. All MLST numbers are from the MLST database. C, clinical; CC, clonal complex; DST, diploid sequence type; E, environmental; FLC, fluconazole; MIC, minimum inhibitory concentration; NTUH, National Taiwan University Hospital; –, not available.

Appendix Table 3. Characteristics and underlying conditions of patients with fluconazole-susceptible and fluconazole-nonsusceptible *Candida tropicalis* bloodstream infections, Taiwan, 2011–2017*

Characteristic	Total, n = 344	With FS *C. tropicalis* BSIs, n = 286	With FNS *C. tropicalis* BSIs, n = 58	p value
Demographics				
Age, y, median (IQR)	62.8 (53.2–73.5)	62.4 (53.0–74.3)	63.4 (55.2–72.1)	0.85
Sex, no. (%)				0.54
M	201 (58.4)	165 (57.7)	36 (62.1)	
F	143 (41.6)	121 (42.3)	22 (37.9)	
Underlying conditions				
Charlson comorbidity index, median (IQR)	4.0 (2.0–6.0)	3.5 (2.0–6.0)	4.0 (2.0–7.0)	0.95
Condition, no. (%)				
Myocardial infarction	37 (10.8)	30 (10.5)	7 (12.1)	0.65
Congestive heart failure	29 (8.4)	25 (8.7)	4 (6.9)	0.80
Peripheral occlusive arterial disease	1 (0.3)	1 (0.3)	0	0.99
Cerebrovascular diseases	24 (7.0)	20 (7.0)	4 (6.9)	0.99
Hemiplegia	5 (1.5)	4 (1.4)	1 (1.7)	0.99
Dementia	7 (2.0)	7 (2.4)	0	0.61
Chronic pulmonary disease	17 (4.9)	15 (5.2)	2 (3.4)	0.75
Connective tissue disease	16 (4.7)	14 (4.9)	2 (3.4)	0.99
Peptic ulcer disease	33 (9.6)	28 (9.8)	5 (8.6)	0.99
Mild liver disease	51 (14.8)	45 (15.7)	6 (10.3)	0.42
Liver disease (moderate–severe)	19 (5.5)	13 (4.6)	6 (10.3)	0.11
Renal diseases (moderate–severe)	101 (29.4)	83 (29.0)	13 (28.3)	0.76
Diabetes mellitus without end organ damage	76 (22.1)	60 (21.0)	16 (27.6)	0.27
Diabetes mellitus with end organ damage	3 (0.9)	3 (1.1)	0	0.99
Solid tumor without metastases	95 (27.6)	79 (27.6)	16 (27.6)	0.99
Metastatic solid tumor	83 (24.1)	72 (25.2)	11 (19.0)	0.31
Hematological malignancy				
Leukemia	51 (14.8)	38 (13.3)	13 (22.4)	0.07
Lymphoma	21 (6.1)	18 (6.3)	3 (5.2)	0.99
AIDS	2 (0.6)	1 (0.4)	1 (1.7)	0.31
Primary focus of infection, no. (%)				
Catheter-related bloodstream infection	129 (37.5)	111 (38.8)	18 (31.0)	0.27
Primary fungemia	145 (42.2)	122 (42.7)	23 (39.7)	0.67
Urinary tract infection	64 (18.6)	51 (17.8)	13 (22.4)	0.41
Intraabdominal infection	12 (3.5)	10 (3.5)	2 (3.5)	0.99
Microbiological characteristics				
Time to positivity, d, median (IQR)	18.5 (12.7–22.2)	18.5 (12.7–22.4)	18.8 (13.8–21.5)	0.76
Recent *Candida* colonization, no. (%)	149 (43.3)	126 (44.1)	23 (39.7)	0.54
Concomitant bacteremia, no. (%)	67 (19.5)	60 (21.0)	7 (12.1)	0.15
Other outcome variables, no. (%)				
Deep-seated infections	22 (6.4)	18 (6.3)	4 (6.9)	0.77
LOS after candidemia onset, d; median (IQR)	21.5 (8.0–39.0)	21.0 (8.0–38.0)	22.5 (10.0–46.0)	0.42
Duration of persistence, d; mean ± SD	3.8 ± 6.5	3.7 ± 6.2	4.4 ± 7.8	0.46

*BSIs, bloodstream infections; FNS, fluconazole nonsusceptible; FS, fluconazole susceptible; IQR, interquartile range; LOS, length of stay.

Appendix Table 4. Comparisons of clinical and microbiological characteristics among patients with candidemia infected by fluconazole-susceptible *Candida tropicalis* isolates, fluconazole-nonsusceptible isolates belonging to clonal complex 3 (CC3) and other CCs*

Characteristic	With FNS CC3 *C. tropicalis* BSIs (n = 36)	With FNS other CCs *C. tropicalis* BSIs FNS (n = 19)	With FS *C. tropicalis* BSIs (n = 110)	p value
Demographics				
Age, y; median (IQR)	61.7 (56.3–68.4)	66.7 (54.5–74.7)	65.2 (53.8–75.0)	0.60
Sex, no. (%)				0.61
M	23 (63.9)	12 (63.2)	61 (55.5)	
F	13 (36.1)	7 (36.8)	49 (44.5)	
Comorbid conditions				
Charlson comorbidity index, median (IQR)	4.5 (3.0–7.0)	2.0 (1.0–4.0)	3.0 (2.0–6.0)	**0.006**†
Myocardial infarction	1 (2.8)	5 (26.3)	9 (8.2)	**0.02**†,‡
Congestive heart failure	2 (5.6)	1 (5.3)	8 (7.3)	0.99
Peripheral occlusive arterial disease	0	0	1 (0.9)	0.99
Cerebrovascular diseases	3 (8.3)	1 (5.3)	7 (6.4)	0.89
Hemiplegia	0	1 (5.3)	1 (0.9)	0.26
Dementia	0	0	4 (3.6)	0.74
Chronic pulmonary disease	2 (5.6)	0	7 (6.4)	0.76
Connective tissue disease	1 (2.8)	1 (5.3)	6 (5.5)	0.99
Peptic ulcer	4 (11.1)	1 (5.3)	12 (10.9)	0.86
Liver disease, mild	6 (16.7)	0	10 (9.1)	0.12
Liver disease, moderate–severe	4 (11.1)	2 (10.5)	5 (4.6)	0.23
Renal disease, moderate–severe	12 (33.3)	4 (21.1)	39 (35.5)	0.53
Diabetes mellitus without end organ damage	11 (30.6)	5 (26.3)	24 (21.8)	0.55
Diabetes mellitus with end organ damage	0	0	1 (0.9)	0.99
Solid tumor without metastases	9 (25.0)	5 (26.3)	26 (23.6)	0.96
Metastatic solid tumor	10 (27.8)	1 (5.3)	28 (25.5)	0.11
Leukemia	10 (27.8)	3 (15.8)	17 (15.5)	0.25
Lymphoma	3 (8.3)	0	5 (4.1)	0.53
Acquired immunodeficiency syndrome	1 (2.8)	0	1 (0.9)	0.56
Healthcare factors, no. (%)§				
Solid organ transplant	1 (2.9)	0	1 (0.9)	0.55
Hematopoietic stem cell transplant	1 (2.9)	0	4 (3.6)	0.99
Major surgery	2 (5.6)	3 (15.8)	13 (11.8)	0.47
Parenteral hyperalimentation	24 (66.7)	9 (47.4)	59 (53.6)	0.29
Steroid use	26 (72.2)	11 (57.9)	36 (32.7)	**<0.001**¶
Chemotherapy	24 (66.7)	5 (26.3)	46 (41.8)	**0.007**†,¶
Neutropenia	15 (42.9)	6 (31.6)	23 (21.5)	**0.050**
Mechanical ventilator	9 (25.0)	6 (31.6)	30 (27.3)	0.90
Indwelling urinary catheter	18 (50.0)	9 (47.4)	38 (34.6)	0.20
Central venous catheter	30 (83.3)	15 (79.0)	96 (87.3)	0.50
Antifungal drug exposure	24 (66.7)	2 (10.5)	13 (11.8)	**<0.001**†,¶
Antimicrobial drug exposure	35 (97.2)	15 (79.0)	96 (87.3)	0.07
Disease severity				
ICU onset, no. (%)	11 (30.6)	8 (42.1)	33 (30.0)	0.58
APACHE II score, median (IQR)	19.5 (16.0–26.0)	17.0 (14.0–23.0)	19.0 (16.0–26.0)	0.59
Primary focus of infection, no. (%)				
Catheter-related bloodstream infection	11 (30.6)	7 (36.8)	51 (46.4)	0.22
Primary fungemia	10 (27.8)	10 (52.6)	38 (34.5)	0.19
Urinary tract infection	10 (27.8)	3 (15.8)	23 (20.9)	0.60
Intraabdomenal infection	2 (5.6)	0	4 (3.6)	0.67
Microbiological characteristics, no. (%)				
Time to positivity, d; median (IQR)	17.7 (13.8–21.5)	19.0 (16.7–20.1)	16.8 (13.0–21.2)	0.47
Recent *Candida* colonization	18 (50.0)	5 (26.3)	51 (46.4)	0.23
Concomitant bacteremia	4 (11.1)	3 (15.8)	23 (20.9)	0.43
Therapeutic intervention, no. (%)#				
Early appropriate antifungal agents	17 (47.2)	1 (5.3)	96 (87.3)	**<0.001**†,‡,¶
Fluconazole as the first antifungal agent	20 (55.6)	15 (79.0)	75 (68.2)	0.20
Early removal of central venous catheter	17/30 (56.7)	11/15 (73.3)	50/96 (52.1)	0.30
Clinical outcomes, no. (%)				
Deep-seated infections	3 (8.3)	1 (5.3)	9 (8.2)	0.99
Death				
7 d	8 (22.2)	3 (15.8)	15 (13.6)	0.46
14 d	11 (30.6)	5 (26.3)	32 (29.1)	0.99
28 d	16 (44.4)	8 (42.1)	51 (46.4)	0.94
In hospital	26 (72.2)	10 (52.6)	71 (64.6)	0.34
LOS after candidemia onset, d; median (IQR)	25.5 (9.5–48.0)	20.0 (14.0–44.0)	23.0 (10.0–39.0)	0.96

Characteristic	With FNS CC3 *C. tropicalis* BSIs (n = 36)	With FNS other CCs *C. tropicalis* BSIs FNS (n = 19)	With FS *C. tropicalis* BSIs (n = 110)	p value
Microbiological outcomes				
Persistence, no. (%)**	11 (35.5)	4 (25.0)	40 (40.4)	0.51
Duration of persistence, d; mean +SD	4.8 + 8.0	3.5 + 7.7	5.7 + 7.6	0.53

*Bold text indicates statistical significance for overall comparison. BSI, bloodstream infection; FNS, fluconazole nonsusceptible; FS, fluconazole susceptible; ICU, intensive care unit; IQR, interquartile range; LOS, length of stay; SD, standard deviation.
†Pairwise comparisons with Bonferroni corrections were conducted when overall p value <0.05. p value of pairwise comparisons between fluconazole nonsusceptible clonal complex 3 and fluconazole nonsusceptible other clonal complexes <0.05 were listed.
‡Pairwise comparisons with Bonferroni corrections were conducted when overall p value <0.05. p value of pairwise comparisons between fluconazole nonsusceptible other clonal complexes and fluconazole susceptible groups <0.05 were listed.
§Major surgery refers to cardiovascular or abdominal surgery. Classes of antifungal exposure azole or echinocandin, 31/3 in FS group vs. 24/2 in FNS group; of note, 14 (24.1%) patients in FNS group experienced breakthrough bloodstream infections compared with18 (6.3%) patients in FS group (p<0.001).
¶ Pairwise comparisons with Bonferroni corrections were conducted when overall p value <0.05. p value of pairwise comparisons between fluconazole nonsusceptible clonal complex 3 and fluconazole susceptible groups <0.05 were listed.
#Early adequate antifungal agents refers to administration of the recommended dose of an intravenous antifungal agent within 48 h after first positive blood culture collection for a susceptible *Candida* isolate, according to the Clinical and Laboratory Standards Institute (CLSI) species-specific breakpoints (*7*). Early removal of central venous catheters is defined as removal of all similar devices, including tunneled and peripherally inserted central catheters, within 48 h after obtaining the first positive blood culture.
**Persistence is defined as >5 days of blood cultures positive for the same Candida species.

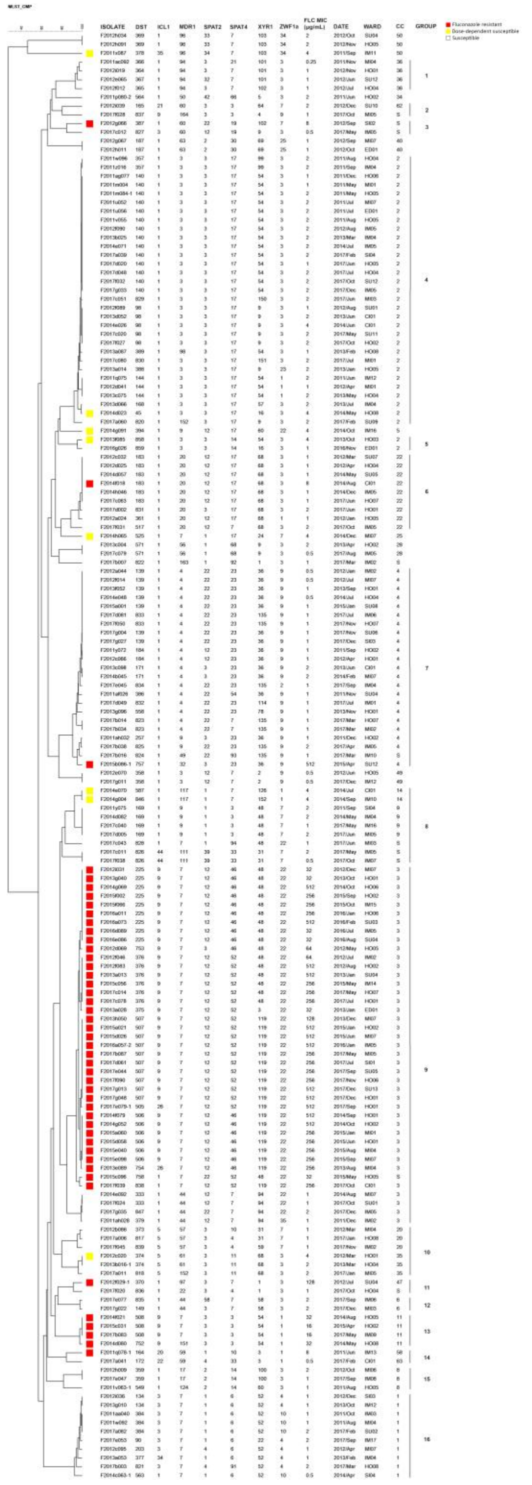

Appendix Figure 1. Dendrogram generated from multilocus sequence typing (MLST) data for 165 non-duplicate *Candida tropicalis* blood isolates, Taiwan, 2011–2017. Phylogenetic analysis by the unweighted pair group method with arithmetic averages**.** Strains with ≥80% similarity are indicated with colored bars and classified as fluconazole resistant (red), dose-dependent susceptible (yellow), or susceptible (white). Wards and number of isolates in ward group from which blood samples were collected are indicated; ED,

emergency department; HO, hemato-oncology; IM, internal medicine; MI, medical intensive care unit; SI, surgical intensive care unit; and SU, surgery. CC, clonal complex; DST, diploid sequence type; FLC, fluconazole. Scale bar indicates the percentage identity.

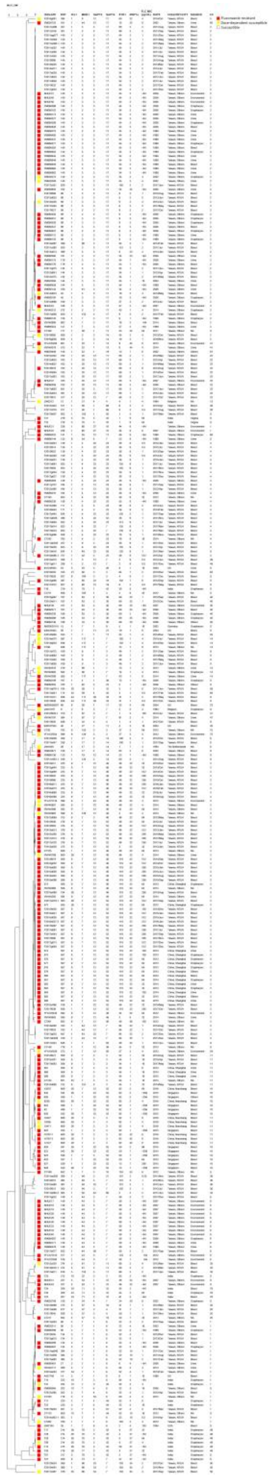

Appendix Figure 2. Dendrogram generated from multilocus sequence typing data for 350 non-duplicate *Candida tropicalis* clinical and environmental isolates. We conducted phylogenetic analysis by the unweighted pair group method with arithmetic averages**.** Isolates are indicated as fluconazole resistant (red), dose-dependent susceptible (yellow), and susceptible (white). CC, clonal complex; DST, diploid sequence type; FLC, fluconazole; NA, not available. Scale bar indicates the percentage identity.

References

1. Jolly K. *Candida tropicalis* MLST central database sited at the University of Oxford. https://pubmlst.org/ctropicalis

2. Wang Y, Shi C, Liu JY, Li WJ, Zhao Y, Xiang MJ. Multilocus sequence typing of *Candida tropicalis* shows clonal cluster enrichment in azole-resistant isolates from patients in Shanghai, China. Infect Genet Evol. 2016;44:418–24. PubMed https://doi.org/10.1016/j.meegid.2016.07.026

3. Chew KL, Cheng JWS, Jureen R, Lin RTP, Teo JWP. *ERG11* mutations are associated with high- level azole resistance in clinical *Candida tropicalis* isolates, a Singapore study. Mycoscience. 2017;58:111–5. https://doi.org/10.1016/j.myc.2016.11.001

4. Lo HJ, Tsai SH, Chu WL, Chen YZ, Zhou ZL, Chen HF, et al. Fruits as the vehicle of drug resistant pathogenic yeasts. J Infect. 2017;75:254–62. PubMed https://doi.org/10.1016/j.jinf.2017.06.005

5. Jin L, Cao Z, Wang Q, Wang Y, Wang X, Chen H, et al. MDR1 overexpression combined with ERG11 mutations induce high-level fluconazole resistance in *Candida tropicalis* clinical isolates. BMC Infect Dis. 2018;18:162. PubMed https://doi.org/10.1186/s12879-018-3082-0

6. Scordino F, Giuffrè L, Barberi G, Marino Merlo F, Orlando MG, Giosa D, et al. Multilocus sequence typing reveals a new cluster of closely related *Candida tropicalis* genotypes in Italian patients with neurological disorders. Front Microbiol. 2018;9:679. PubMed https://doi.org/10.3389/fmicb.2018.00679

7. Clinical and Laboratory Standards Institute. Reference method for broth dilution antifungal susceptibility testing of yeasts: Fourth Informational Supplement M27–S4. Wayne (PA): The Institute; 2012.
